# A teacher with progressive headache, dyslexia, dysgraphia and seizure

**Published:** 2013

**Authors:** Siamak Abdi, Askar Ghorbani

**Affiliations:** 1 Resident, Department of Neurology, School of Medicine AND Shariati Hospital, Tehran University of Medical Sciences, Tehran, Iran; 2 Assistant Professor, Department of Neurology, School of Medicine AND Shariati Hospital, Tehran University of Medical Sciences, Tehran, Iran

**Keywords:** MRI, Brain Mass, Corpus Callosum

## Case

A 56-year-old female teacher presented to Shariati Hospital, Tehran, Iran, with history of progressive headache, difficulty to read and write (dyslexia and dysgraphia), and seizure since 3 months ago. Neurological examination demonstrated mild bilateral papilledema and left side Babinski sign. The exam was otherwise normal.

**Figure 1:**
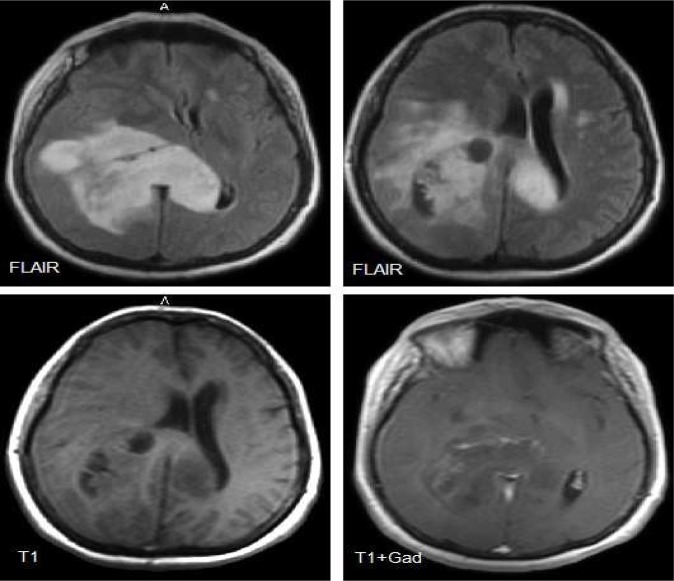
FLAIR images from different levels in axial brain MRI

Brain magnetic resonance imaging (MRI) was performed (Figure 1).

1. What are the imaging findings?
2. What are the probable differential diagnoses?
3. What is the most probable diagnosis?
4. Does the lesion explain dysgraphia and dyslexia?

## Answers

1. The brain MRI showed an ill-defined fluid attenuated inversion recovery (FLAIR) hyperintense/T1 hypointense lesion in right deep parietotemporal lobes extending to contralateral occipital lobe via splenium of corpus callosum causing mass effect and subfalcine herniation. Some small FLAIR hyperintense lesions were also seen in left frontoparietal subcortical area. After contrast administration, non-homogenous enhancement was seen in some parts of the lesion.
2. Mass lesions involving splenium of corpus callosum include glioblastoma multiforme (GBM), anaplastic astrocytoma and primary central nervous system (CNS) lymphoma (PCNSL).
3. Being hyperintense in FLAIR, and non-homogenous faint gad-enhancement (Gadolinium) were in favor of GBM. Anaplastic astrocytoma scan was similar to GBM, but usually did not enhance. PCNSL was usually hypointense in T2 and showed avid enhancement, except in immunosuppressed (e.g. AIDS) patients.^1^
4. Alexia with agraphia is usually seen in left angular gyrus lesions.^2^ Surprisingly, the brain MRI of our patient did not show the left inferior parietal (angular gyrus) involvement. However, GBM was an infiltrative neoplasm, with histologic involvement far beyond visible radiologic tumor. Therefore, it can be concluded that though it was not seen on MRI, inferior parietal lobe was infiltrated by tumor.

## Discussion

Glioblastoma multiform is the most common primary malignant brain tumor.^3^ Presenting symptoms are diverse and related to the site of tumor. Diagnosis was proposed by imaging and established by histopathological study. MRI is used as a standard imaging modality in many centers. However, newer sequences [diffusion weighted imaging (DWI), diffusion tensor imaging (DTI), susceptibility weighted imaging (SWI), and magnetic resonance spectroscopy (MRS)] and modalities such as positron emission tomography (PET) are emerging which can help in determining the diagnosis, prognosis, and treatment plan.^2, 4^ GBM lesions are generally heterogeneously hyperintense in T2/FLAIR and hypo/isointense in T1 sequence. Hemorrhage, necrosis, cyst, fluid/debris level and flow voids (sign of neovascularity) were common findings. Different patterns of enhancement (solid, patchy, ring, and nodular) may be seen.^1^ Surgery has been the mainstay of treatment, but radiotherapy, radiosurgery and new chemotherapeutic agents have been introduced as adjuvant or alternative treatment options.^2, 5^
